# Unveiling the tradeoff between device scale and surface nonidealities for an optimized quality factor at room temperature in 2D MoS_2_ nanomechanical resonators

**DOI:** 10.1038/s41378-024-00763-9

**Published:** 2024-09-27

**Authors:** Pengcheng Zhang, Yueyang Jia, Shuai Yuan, Maosong Xie, Zuheng Liu, Hao Jia, Rui Yang

**Affiliations:** 1 University of Michigan–Shanghai Jiao Tong University Joint Institute, Shanghai Jiao Tong University, Shanghai, 200240 China; 2 Shanghai Institute of Microsystem and Information Technology, Chinese Academy of Sciences, Shanghai, 200050 China; 3 State Key Laboratory of Radio Frequency Heterogeneous Integration, Shanghai Jiao Tong University, Shanghai, 200240 China

**Keywords:** NEMS, Electrical and electronic engineering

## Abstract

A high quality (*Q*) factor is essential for enhancing the performance of resonant nanoelectromechanical systems (NEMS). NEMS resonators based on two-dimensional (2D) materials such as molybdenum disulfide (MoS_2_) have high frequency tunability, large dynamic range, and high sensitivity, yet room-temperature *Q* factors are typically less than 1000. Here, we systematically investigate the effects of device size and surface nonidealities on *Q* factor by measuring 52 dry-transferred fully clamped circular MoS_2_ NEMS resonators with diameters ranging from 1 μm to 8 μm, and optimize the *Q* factor by combining these effects with the strain-modulated dissipation model. We find that *Q* factor first increases and then decreases with diameter, with an optimized room-temperature *Q* factor up to 3315 ± 115 for a 2-μm-diameter device. Through extensive characterization and analysis using Raman spectroscopy, atomic force microscopy, and scanning electron microscopy, we demonstrate that surface nonidealities such as wrinkles, residues, and bubbles are especially significant for decreasing *Q* factor, especially for larger suspended membranes, while resonators with flat and smooth surfaces typically have larger *Q* factors. To further optimize *Q* factors, we measure and model *Q* factor dependence on the gate voltage, showing that smaller DC and radio-frequency (RF) driving voltages always lead to a higher *Q* factor, consistent with the strain-modulated dissipation model. This optimization of the *Q* factor delineates a straightforward and promising pathway for designing high-*Q* 2D NEMS resonators for ultrasensitive transducers, efficient RF communications, and low-power memory and computing.

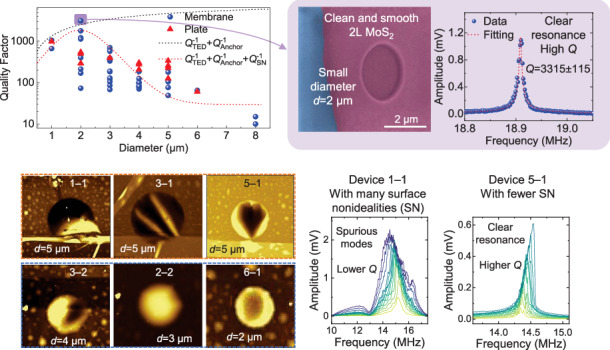

## Introduction

The quality (*Q*) factor is critical in resonant micro/nanoelectromechanical systems (MEMS/NEMS) because a higher *Q* factor represents lower energy dissipation, better frequency selectivity, and higher sensitivity, all of which are highly desirable for ultrasensitive resonant transducers, low-phase-noise voltage-controlled oscillators, highly selective filters, and ultralow-power memory and computing^1,2^. As resonators scale down to nanoscales and even atomic scales, dissipation mechanisms can become more complex, and it has been reported that the *Q* factor generally decreases with device volume and critical dimension^3^. Specifically, for doubly clamped beam resonators the *Q* factor increases with length but decreases with width^4^. In NEMS resonators, surface loss, anchor loss, frequency-independent material friction, and thermoelastic dissipation have all been considered potential dominant damping mechanisms^5,6^. Therefore, a deep understanding of the size effect on the *Q* factor and effective control of damping are increasingly important.

As resonator size continues to scale down, NEMS resonators based on two-dimensional (2D) materials, such as molybdenum disulfide (MoS_2_), have attracted tremendous interest because they represent the ultimate scaling in the thickness direction and have shown a number of intriguing properties^7^, such as a resonance frequency of up to 1.17 GHz^8^, an ultrawide frequency tuning range of up to 366%^9^, and a large dynamic range of up to 102 dB^10,11^. These 2D NEMS resonators have shown high potential for mass sensing with resolutions reaching ~26 yg^12,13^, force sensing with a sensitivity of 390 zN/Hz^1/2^^14^, highly tunable voltage-controlled oscillators^15,16^, ultralow-power memory and computing^17,18^, coupling with other physical domains^19–21^, and quantum engineering^22–24^. Toward these applications and scientific explorations, a high *Q* factor is desirable for enhancing device performance. While the *Q* factor up to 1 million has been demonstrated for a graphene NEMS resonator at 15 mK^25^, the room-temperature *Q* factors for 2D NEMS resonators remain relatively lower compared with mainstream MEMS resonators, with *Q* factors typically in the range of a few hundred^26–28^ and no more than 2400 demonstrated for 2D NEMS resonators^10,29,30^. Strain, vibration amplitude, mode coupling, and interlayer friction have been reported to be important for their dissipation characteristics^31–35^. The geometric shape and critical dimension can also have important effects on the *Q* factor for 2D NEMS resonators. Theoretical and experimental studies have shown that 2D NEMS resonators with free edges, such as doubly clamped resonators, have larger energy dissipation and smaller *Q* factors than those without free edges such as fully clamped circular drumhead resonators^29,36,37^. Therefore, performing a comprehensive analysis of the damping mechanisms and thoroughly studying the effects of size on damping are critical for optimizing *Q* factors at room temperature for 2D NEMS resonators.

In this work, we systematically investigate the effect of device size on *Q* factors by measuring 52 MoS_2_ NEMS resonators with diameters varying from 1 μm to 8 μm. The results demonstrate that the *Q* factor first increases with diameter from 1 μm to 2 μm then decreases with diameter when larger than 2 μm. This *Q vs*. diameter relationship shows a different trend from previous reports on graphene NEMS resonators in which the *Q* factor monotonically increases with increasing diameter^29^. Through detailed characterization using atomic force microscopy (AFM), scanning electron microscopy (SEM), and Raman spectroscopy, we demonstrate that devices with larger diameters are more likely to include undesirable surface wrinkles, residues, and bubbles on the suspended membrane, which can lead to lower *Q* factors. We further find that as the diameter increases the resonance peaks do not have regular peak shapes and spurious modes emerge. By properly designing device structures as fully clamped circular membranes with 2 μm diameters and optimizing driving conditions based on the strain-modulated dissipation model, we achieve a *Q* factor up to 3315 ± 115 at room temperature for a bilayer MoS_2_ circular drumhead NEMS resonator. These results provide clear guidelines for enhancing the *Q* factor of 2D NEMS resonators at room temperature and pave the way for a number of applications that require high-*Q* NEMS resonators.

## Results and discussion

We fabricate 2D NEMS resonators by first lithographically patterning substrates with surface microtrenches and contact electrodes and then transferring 2D MoS_2_ onto surface microtrenches using a dry-transfer process based on a polydimethylsiloxane (PDMS) stamp, which is widely used in 2D device transfer, especially for fabricating suspended devices^9,20,21,31,32,34,38^. All 2D MoS_2_ membranes are exfoliated from the same piece of bulk material; thus, we assume that minimal variations in material properties exist. The DC gate voltage *V*_GS_ and radio-frequency (RF) driving voltage *v*_RF_ are applied to the gate electrode through a bias tee to capacitively drive the membrane, with the contact electrode grounded. The suspended MoS_2_ membrane is pulled down toward the gate electrode by the electrostatic force induced by *V*_GS_, which leads to tension and resonance frequency tuning. The resonances are measured using a custom-built optical interferometry setup (Supporting Information Section S1)^31,39,40^. To minimize the effects of air damping and evacuate most of the air within the cavity^41,42^, all measurements are carried out after the 2D NEMS resonators are placed in a vacuum chamber (Fig. S1) for a minimum of 1 day. All resonance measurements are performed at a vacuum pressure of 1.2 × 10^−2 ^Torr at a room temperature of 300 K.

**Fig. 1:**
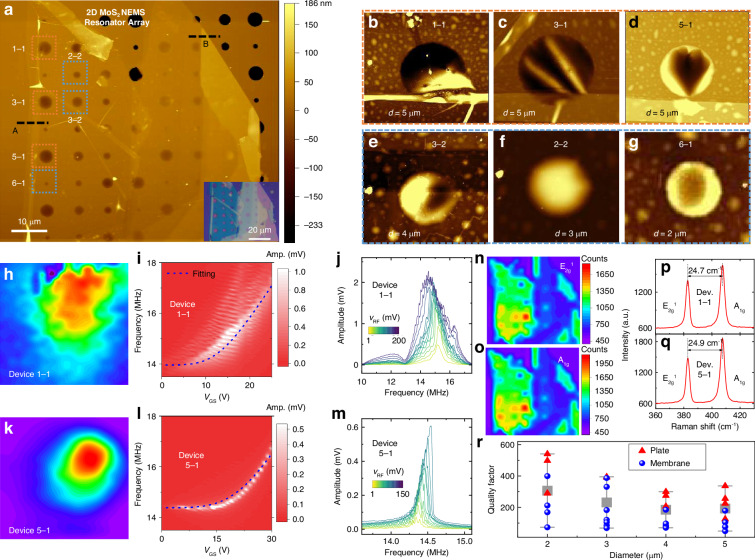
Comparison of characteristics among an array of 2D MoS_2_ NEMS resonators with varying diameters from 2 μm to 5 μm, with either uniform or nonuniform membrane profiles. **a** AFM color maps of the array of resonators. The thin region shown by the black dashed line “A” has a thickness of 5.5 nm (Fig. S2d), and the thick region shown by the black dashed line “B” has a thickness of 19 nm (Fig. S2e). *Inset*: optical image. The zoomed-in AFM color maps of representative resonators with diameters of (**b**–**d**) 5 μm, (**e**) 4 μm, (**f**) 3 μm, and (**g**) 2 μm. Resonance measurements of Device “1–1” with wrinkles on the surface and with a diameter of 5 μm, including (**h**) mapping of the fundamental flexural mode shape, (**i**) resonances at varying *V*_GS_ and a fixed *v*_RF_ of 10 mV with the amplitude shown in a color scale, and (**j**) resonances at varying *v*_RF_ with a fixed *V*_GS_ of 10 V. In the resonator named “*m*–*n*”, *m* indicates the row number and *n* indicates the column in the array. Measurements of Device “5–1” with fewer wrinkles on the surface and with a diameter of 5 μm, including (**k**) mapping of the fundamental flexural mode shape, (**l**) resonances at varying *V*_GS_ and a fixed *v*_RF_ of 10 mV, with the amplitude shown in a color scale, and (**m**) resonances at varying *v*_RF_ with a fixed *V*_GS_ of 10 V. Raman mapping of the resonator array with the color showing the Raman peak amplitude for the (**n**) E_2g_^1^ peak and (**o**) A_1g_ peak. Raman spectra of Devices (**p**) 1–1 and (**q**) 5–1 measured near the center of the suspended membrane. **r** Summarized *Q* factors of the resonator array and modeled *Q* factor *vs*. diameter relationship, with diameters ranging from 2 μm to 5 μm. We plot the highest *Q* factor for each device when varying the gate voltage. The red triangles are the *Q* factors for devices in the plate regime (thickness >10 nm) and the blue dots are the *Q* factors for devices in the membrane regime (thickness ≤10 nm)

To measure the *Q* factors at different diameters while minimizing the effects of the variation in the MoS_2_ material, we fabricate an array of 2D MoS_2_ NEMS resonators on a substrate with 8 × 8 circular microtrenches of various diameters (Fig. 1a). The fully clamped resonator structure avoids the undesirable effects of free edges on energy dissipation and can enhance the *Q* factor^29,36^. We measure resonances from 34 resonators in the array and name each device as Device “*m*–*n*”, where *m* is the row number and *n* is the column number. The membrane surface is first characterized using AFM, showing the surface quality of the suspended membranes (Fig. 1a). Comparisons show that resonators with larger diameters (>3 μm) typically contain more wrinkles (Fig. 1b–e) on the suspended membranes than those with smaller diameters (2–3 μm) (Fig. 1f, g). We perform Raman mapping to measure the uniformity of the MoS_2_ material (Fig. 1n–q), where, for MoS_2_ with the same thickness, uniformly suspended MoS_2_ membranes generally show slightly higher Raman peak intensities than wrinkled membranes. The resonance spectrum for each resonator is recorded and fitted to the solution for the equation of motion to extract the *Q* factor (Fig. S4), with the *Q* factors summarized in Fig. 1r. We find that the *Q* factors can vary by several times for different devices with the same diameter and thickness, i.e., from 49 (Device 1–1) to 179 (Device 5–1). Furthermore, the *Q* factors of thicker resonators in the plate regime are typically slightly higher than those of thin resonators in the membrane regime. We then perform in-depth resonance measurements for two representative resonators (Devices 1–1 and 5–1). Mode mapping measurements are performed by fixing the laser and scanning the stage that holds the vacuum chamber with the resonator inside, demonstrating that Device 1–1, with more wrinkles on the surface (Fig. 1b), also show spurious modes (Fig. 1i–j) and a nonuniform mode shape (Fig. 1h). In contrast, Device 5–1, with a more uniform surface and fewer wrinkles (Fig. 1d), clearly shows a fundamental flexural mode shape (Fig. 1k) and regular frequency tuning characteristics (Fig. 1l). By gradually increasing the *v*_RF_, Device 5–1 exhibits linear to Duffing nonlinear resonances with hardening (Fig. 1m). The gate-tunable resonance frequency model can fit the frequency tuning characteristics of Device 5–1 well but not those of Device 1–1 because of the spurious modes (Supporting Information Section S4.1–S4.2), as follows^31^:

1 $${f}_{res}=\frac{1}{2\pi }\sqrt{\frac{{2.405}^{4}{E}_{Y}{\varepsilon }_{r}}{2\rho {R}^{2}}-\frac{{\varepsilon}_{0}}{0.75\rho{t}{g}^{3}}{{V}_{GS}}^{2}}$$

where *R* is the radius, *t* is the thickness, *E*_Y_ is the Young’s modulus, *ν* is the Poisson’s ratio, *ε*_r_ is the gate-tunable total strain, *g* is the initial vacuum gap, *ϵ*_0_ is the vacuum permittivity, and *ρ* is the mass density.

The lower *Q* factors for devices with more wrinkles and residues on the surface suggest the importance of surface-induced damping mechanisms in these 2D NEMS resonators. From the summarized *Q vs*. diameter relationship in Fig. 1r, we find that the *Q* factor generally decreases with increasing diameter in the range of 2 μm to 5 μm. If anchor loss or thermoelastic damping dominates, then according to previous models^32,43^, the *Q* factor should increase with increasing resonator diameter. Therefore, the results in Fig. 1r suggest that as the resonator size increases there is a higher chance that surface residues and wrinkles induce more energy loss.

**Fig. 2:**
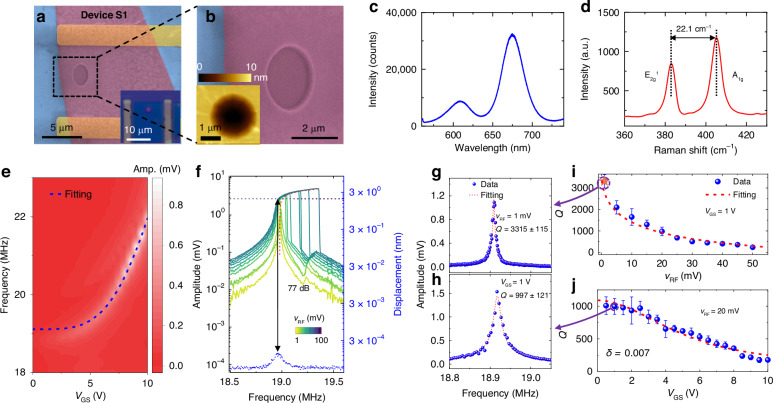
Resonance characteristics for another MoS_2_ NEMS resonator (Device S1) with a diameter of 2 μm, which is a singly-isolated resonator with a bilayer thickness and flat, smooth suspended material. **a** False-colored SEM image of the resonator. *Inset*: optical image. **b** Zoomed-in false-colored SEM image of the resonator. *Inset*: AFM image. **c** PL spectrum showing high intensity. **d** Raman spectrum showing the difference between the E_2g_^1^ and A_1g_ peaks at ~22.1 cm^−1^, corresponding to bilayer MoS_2_. Measured resonances (**e**) at varying *V*_GS_ at a fixed *v*_RF_ of 20 mV, with the amplitude shown in color scale, and (**f**) at varying *v*_RF_ at a fixed *V*_GS_ of 1 V, with the dynamic range of 77 dB extracted^12^. Representative resonance spectra at (**g**) *V*_GS_ = 1 V and *v*_RF_ = 1 mV, with a *Q* factor up to 3315 ± 115, and (**h**) *V*_GS_ = 1 V and *v*_RF_ = 20 mV, with a *Q* factor of 997 ± 21. Summary of extracted *Q* factors at varying (**i**) *v*_RF_ and (**j**) *V*_GS_ values and fitting to the strain-modulated dissipation model, with a *δ* of 0.007 extracted

To further validate the size dependence of *Q* factor for thinner resonators and to achieve a high *Q* factor in MoS_2_ NEMS resonators, we fabricate another singly-isolated MoS_2_ NEMS resonator (Device S1) with a diameter of 2 μm and a bilayer thickness. The clean, flat, and smooth surface in the suspended region is confirmed by the SEM images (Fig. 2a, b). In addition, the bilayer thickness and high quality of the MoS_2_ material are confirmed by the high PL intensity and Raman peak separation of 22.1 cm^−1^ (Fig. 2c, d)^39,44^. *V*_GS_ tuning of the resonance frequency shows a trend that can be well fitted by the frequency tuning model (Fig. 2e), and resonance measurements obtained by increasing the *v*_RF_ clearly reveal a transition from undriven thermomechanical resonance to nonlinear driven resonances (Fig. 2f). Furthermore, the *Q* factor decreases with increasing *V*_GS_ and *v*_RF_ (Fig. 2g–j), which can be well fitted to the strain-modulated thermoelastic dissipation model (Supporting Information Section S4.3):

2 $${Q}_{TED}^{-1}=\frac{5.576{{x}_{0}}^{2}}{{\varepsilon }_{r}({V}_{GS}){R}^{2}(1-{v}^{2})}\delta ,$$

where *δ* is a fitting parameter representing the loss angle in the complex form of Young's modulus, and *x*_0_ is the vibration amplitude that is proportional to |*V*_GS _× *v*_RF_|. From fitting to the gate tuning of the *Q* factor we can extract *δ* for each resonator. By optimizing the diameter, minimizing surface nonidealities, and decreasing the driving strength, we achieve a high *Q* factor up to 3315 ± 115 at *v*_RF_ = 1 mV and *V*_GS_ = 1 V (Fig. 2g). This *Q* factor is comparable to that of several piezoelectric MEMS resonators measured under similar conditions^45^.

**Fig. 3:**
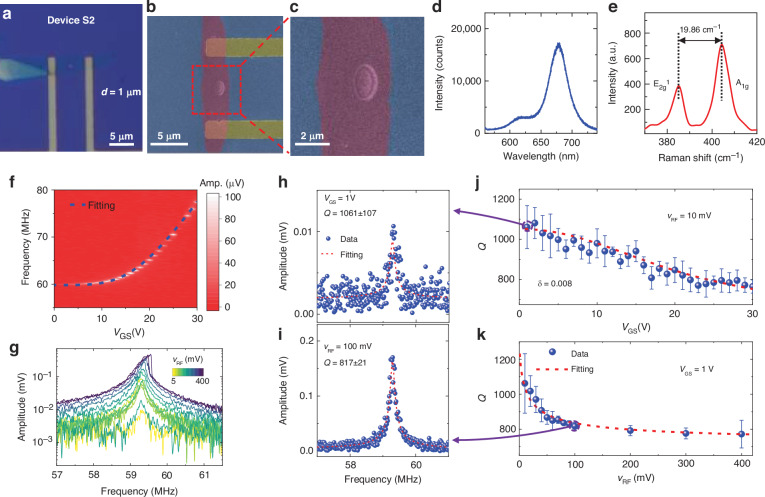
Gate-tunable characteristics for a monolayer MoS_2_ circular drumhead resonator (Device S2) with flat suspended material and without observable surface nonidealities. **a** Optical image of the device with a diameter of 1 μm. **b**, **c** False-colored SEM images showing the flat suspended membrane. Measured (**d**) photoluminescence spectrum with high intensity and (**e**) Raman spectrum with a difference between the E^1^_2g_ and A_1g_ peaks of 19.86 cm^−1^, corresponding to monolayer MoS_2_. **f** Frequency tuning characteristics measured at a fixed *v*_RF_ of 10 mV, with the amplitude shown in color scale. **g** Linear to nonlinear resonances obtained by increasing the *v*_RF_ at a fixed *V*_GS_ of 1 V. Extracted *Q* factors and modeled dependence on (**j**) *V*_GS_ and (**k**) *v*_RF_, showing typical resonances at (**h**) *V*_GS_ = 1 V with a *Q* factor of 1061 ± 107 and (**i**) *v*_RF_ = 100 mV with a *Q* factor of 817 ± 21. From the gate-tunable *Q* factor, the loss angle *δ* of 0.008 is extracted from fitting *via* the strain-modulated dissipation model

To study the *Q vs*. diameter relationship more comprehensively, we further scale down the resonators and measure a singly-isolated resonator, Device S2, with a 1 μm diameter (Fig. 3a) and monolayer thickness (Fig. 3d, e). The flat and smooth surface is confirmed by the SEM images (Fig. 3b, c) which show no observable residue or wrinkle. From the measured resonances (Fig. 3f, g), we extract *Q* factor at each *V*_GS_ and *v*_RF_ and obtain a *Q* factor up to 1051 ± 77 (Fig. 3h–k). By comparing the results from Figs. 2 to 3 we find that when the resonator surfaces are clean, the resonators with larger diameters have larger *Q* factors.

**Fig. 4:**
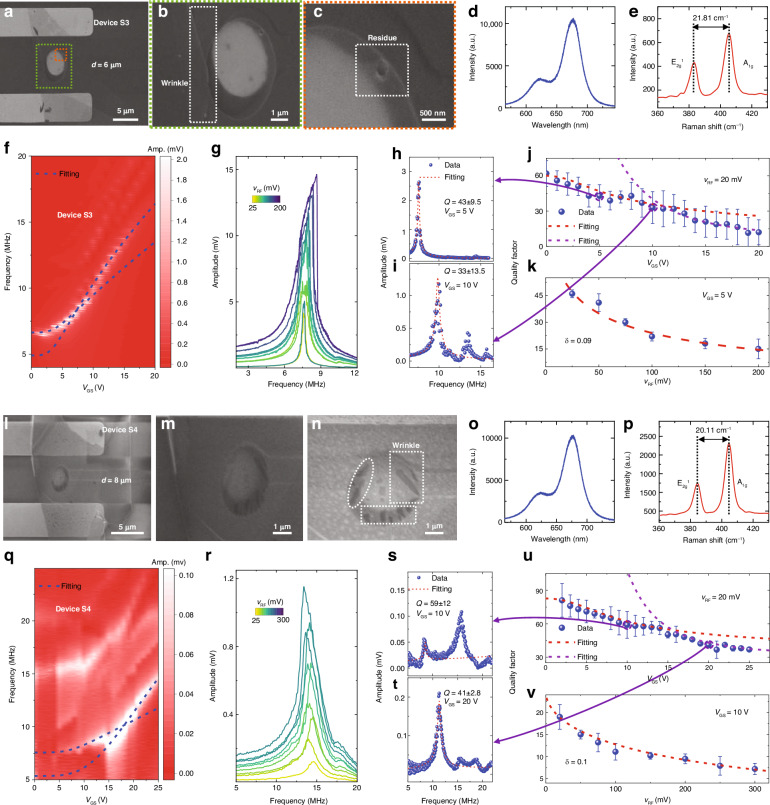
Gate tuning of frequency and dissipation characteristics and material characterization for two singly-isolated 2D MoS_2_ drumhead NEMS resonators with nonuniform membrane profiles. **a**–**k** Characterization of Device S3. SEM images of Device S3 showing (**a**) the suspended membrane diameter of 6 μm with contact electrodes, (**b**) the zoom-in region in (**a**) showing the wrinkle, and (**c**) the zoom-in region in (**b**) showing the residue on the suspended membrane. **d** Photoluminescence spectrum. **e** Raman spectrum showing that the peak separation between the E_2g_^1^ and A_1g_ peaks is 21.81 cm^−1^, corresponding to bilayer MoS_2_. **f** Frequency tuning characteristics measured by varying *V*_GS_ from 0 V to 20 V with a fixed *v*_RF_ of 20 mV, with the amplitude shown in color scale. **g** Measured linear to nonlinear resonances by varying *v*_RF_ from 25 mV to 200 mV with a fixed *V*_GS_ of 5 V. **h**–**k** Extraction of *Q* factors at varying gate voltages, showing (**h**) fitting to the resonance at *V*_GS_ = 5 V, and (**i**) fitting to the resonance at *V*_GS_ = 10 V. Summary of the measured *Q* factor and fitting to the dissipation model at varying (**j**) *V*_GS_, and (**k**) *v*_RF_, from which *δ* of 0.09 is extracted. **l**–**v** Measured characteristics of Device S4, with a diameter of 4 μm, shown in the same sequence as in (**a**–**k**). The SEM images in (**l**–**n**) also show some wrinkles. The measured Raman peak separation of 20.11 cm^−1^ in (**p**) corresponds to monolayer MoS_2_. From fitting to the measured gate tuning of the *Q* factor, a *δ* of 0.1 is extracted

To further investigate the relationship between the *Q* factor and diameter, we measure 2D MoS_2_ NEMS resonators with larger diameters. For resonator Device S3 with a diameter of 6 μm (Fig. 4a) and bilayer thickness (Fig. 4d, e), the zoomed-in SEM images clearly show wrinkles (Fig. 4b) and residues (Fig. 4c) close to the clamping ends. From resonance measurements we find that the frequency tuning characteristics before and after *V*_GS_ = 10 V show different increasing trends (Fig. 4f). The mode shapes, frequency tuning characteristics, and resonance curves of MoS_2_ NEMS resonators with surface residues or wrinkles under different damping conditions are simulated using the finite element method (FEM) (Figs. S18–S20), with abnormal mode shapes for the fundamental flexural mode observed. For the resonator with residue, we further demonstrate that the mode shape and the position of the maximum vibration amplitude change when the gate voltage increases (Fig. S18). The irregular mode shape at each *V*_GS_ determines the dynamic energy in the device, which could result in nonideal frequency tuning characteristics and larger dissipation. The extracted *Q* factors at each *V*_GS_ and *v*_RF_ indicate that the *Q* factors are low and that *Q* decreases with increasing *V*_GS_ and *v*_RF_ (Fig. 4h–k). Fitting to the *Q vs. V*_GS_ relationship also shows a turning point before and after *V*_GS_ = 10 V, similar to the frequency tuning characteristics. The low *Q* factor and nonideal characteristics for gate tuning of *Q* factor suggest correlations with the dynamic-energy-dependent loss angle *δ* (Supporting Information Section S4 and Table S1), with resonators with larger diameters and lower *Q* factors generally having higher *δ* values. To confirm this effect, we further measure the frequency tuning characteristics of another device (Device S8) with the same 6-μm diameter (Fig. S7), which also has relatively low *Q* factors and nonideal resonance peak shapes.

For another MoS_2_ NEMS resonator (Device S4) with an even larger diameter of 8 μm and with a monolayer thickness (Fig. 4o, p), the SEM images also show that the suspended membrane contains several wrinkles (Fig. 4l–n). The frequency tuning characteristics (Fig. 4q) and Duffing nonlinearity (Fig. 4r) are also measured, but owing to the multiple spurious modes, the frequency tuning trend is not clear and cannot be fitted consistently using the model. Multiple resonance peaks show resonance frequencies similar to those of well-separated resonances, as predicted by the classical model (Fig. 4s, t)^46^, which could also be attributed to surface nonidealities. The nonideal frequency tuning characteristics and mode shapes due to wrinkles are simulated using FEM (Figs. S19–S20). The simulated resonance characteristics show that the spurious resonance modes become more obvious with greater *v*_RF_ driving (Fig. S20). Furthermore, for resonators with small *Q* factors due to surface nonideality and large *v*_RF_, the resonance frequencies can also be close to each other, leading to merged resonances with a small peak for the spurious mode and a small frequency selectivity. These findings are consistent with the results presented in Fig. 1j. A summary of the fitting to all the resonances at various *V*_GS_ and *v*_RF_ also reveals low *Q* factors and decreasing *Q* factors with larger *V*_GS_ and *v*_RF_, which cannot be fitted well above *V*_GS_ = 15 V (Fig. 4u, v). To further confirm the consistency of the effects of diameter and surface nonideality on device properties, we obtain measurement results from 13 additional singly-isolated MoS_2_ drumhead NEMS resonators with various diameters (Devices S6–S18), which consistently show that larger membranes typically contain more surface nonidealities on the suspended membranes and thus have lower *Q* factors (Figs. S5–S17).

**Fig. 5:**
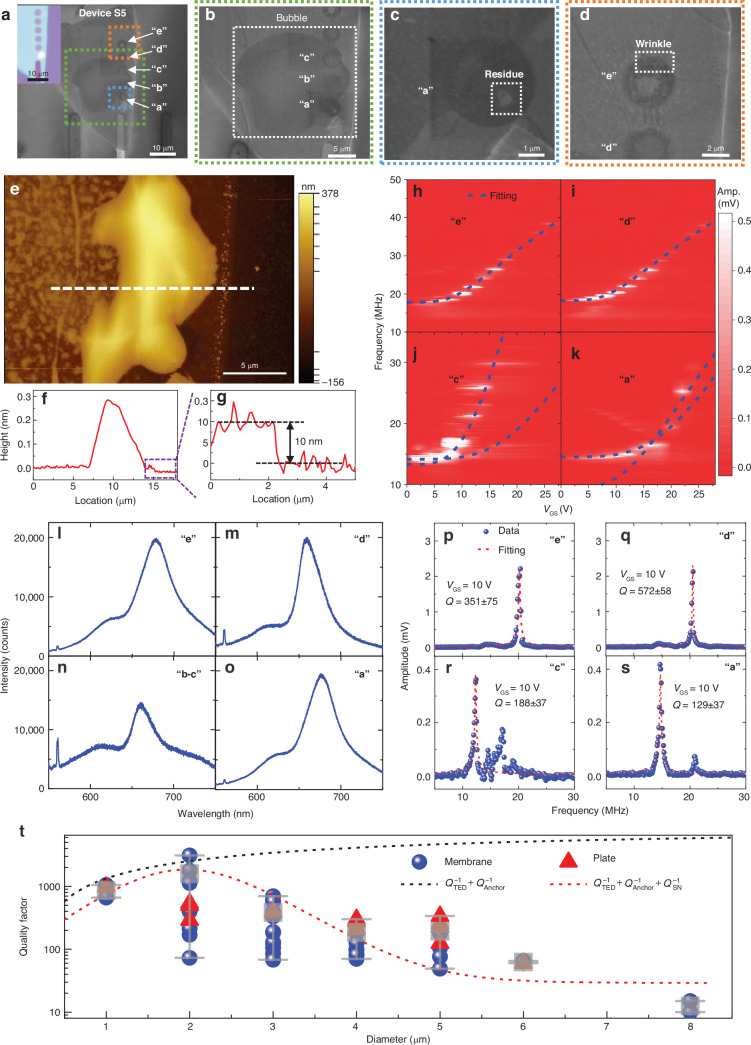
Comparison of characteristics among 2D MoS_2_ NEMS resonators on 5 microtrenches next to each other in Device S5 with a bubble under the material. **a**–**d** SEM images showing (**a**) 5 side-by-side resonators named “a”, “b”, “c”, “d”, and “e” with the same diameter of 3 μm. **b** Resonators “a”, “b”, and “c” with a large bubble outlined by the white dashed box. **c** Resonator “a” with residue on the membrane outlined by the white dashed box. **d** Resonator “e” with wrinkle in the white dashed box. **e** AFM image of the bubble area with the thickness shown in color scale. **f**, **g** Thickness profile measured along the white dashed lines in (**a**), showing (**b**) a maximum height of ~300 nm for the bubble and (**c**) a thickness of 10 nm for the MoS_2_ membrane. Gate tuning of the frequency measured by varying *V*_GS_ from 0 V to 25 V and at a fixed *v*_RF_ of 20 mV for resonators (**h**) “e”, (**i**) “d”, (**j**) “c”, and (**k**) “a”, with the amplitude shown in color scale. Resonator “b” does not show measurable resonance. Photoluminescence spectra measured at different positions for resonators (**l**) “e”, (**m**) “d”, (**n**) “b–c”, and (**o**) “a”. Comparison of typical measured resonances and extracted *Q* factors for different resonators at the same driving force, showing the *Q* factors of (**p**) 351 ± 75 for resonator “e”, (**q**) 572 ± 58 for resonator “d”, (**r**) 188 ± 37 for resonator “c”, and (**s**) 129 ± 37 for resonator “a”, at *V*_GS_ = 5 V. **t** Summarized *Q* factors for all 52 MoS_2_ NEMS resonators and the modeled *Q* factor *vs*. diameter relationship, with diameters ranging from 1 μm to 8 μm. The *Q* factor shown for each device is the largest at varying gate voltages. The black dashed line is the model fitting using *Q*_TED_^−1 ^+ *Q*_Anchor_^−1^ (~*D*^-1.1^)^43^, and the red dashed line is the model fitting using *Q*_TED_^−1 ^+ *Q*_Anchor_^−1^ + *Q*_SN_^−1^ (~*D*^1.2^) + *Q*_Other_^−1^

We further investigate Device S5, with an array of five microtrenches with the same diameter of 3 μm and uniform thickness of 10 nm (Fig. 5a, g) but with a large bubble on the surface formed during device fabrication (Fig. 5b, e). Despite having the same material and geometry, the five resonators in Device S5 have different surface nonidealities on the suspended membrane: resonators “b” and “c” are influenced by the bubble (Fig. 5e), resonator “a” has a residue on the suspended membrane (Fig. 5c), resonator “e” has a small wrinkle near the clamping edge (Fig. 5d), and resonator “d” has a very flat and clean suspended material (Fig. 5g). The PL intensities for resonators “b” and “c” are much lower than those for the other resonators (Fig. 5l–o), further confirming the effects of the bubble. We measure the resonance tuning characteristics of all five devices by varying *V*_GS_. Both resonators “e” and “d” show single clear resonances with high intensity so that the frequency tuning characteristics can be well fitted with our model (Fig. 5h, i). However, resonators “c” and “a” show multiple spurious modes in the resonances (Fig. 5j, k). The extracted *Q* factors for the flat resonator “d” without surface nonidealities are the highest for Device S5 (Fig. 5p–s), which shows that surface bubbles can also lead to larger dissipation. Resonator “e” with a small wrinkle has a lower *Q* factor than resonator “d” but a much higher *Q* factor than resonators “a” and “c”. These control experiments further confirm the importance of surface nonidealities for the resonance characteristics and damping properties of 2D NEMS resonators.

The measurement results from all 52 MoS_2_ NEMS resonators consistently show that devices with large diameters (3–8 μm) generally have irregular resonance frequency tuning characteristics and many spurious modes, especially at large *V*_GS_, due to the higher chance of including surface nonidealities. In contrast, devices with smaller diameters (1–2 μm) show clear resonance modes with frequency and *Q* factor tuning characteristics well fitted by the models. We summarize the measured *Q* factor *vs*. diameter relationship in Fig. 5t, where the *Q* factor for each device is the largest *Q* factor at varying gate voltages. Because dissipation increases with a larger drive (|*V*_GS _× *v*_RF_|), the largest *Q* factor is usually obtained near the first measurable resonance at a small driving amplitude. The *Q* factor first increases and then decreases with increasing diameter, reaching a maximum value at 2 μm and a minimum value at 8 μm. We model the overall effect from multiple damping mechanisms as follows:

3 $${Q}^{-1}={Q}_{TED}^{-1}+{Q}_{Anchor}^{-1}+{Q}_{SN}^{-1}+{Q}_{Other}^{-1},$$

where *Q*_TED_^−1^ represents thermoelastic dissipation, *Q*_Anchor_^−1^ represents anchor loss, *Q*_SN_^−1^ represents the loss induced by surface nonidealities, and *Q*_Other_^−1^ represents other diameter-independent damping mechanisms including air/fluid damping, material-defect-induced damping, and phonon‒phonon dissipation. Surface-induced energy dissipation has been found to limit or decrease the *Q* factor as the device size increases^47,48^. For thermoelastic dissipation and anchor loss, a larger diameter can lead to a larger *Q* factor. When the diameter is 1–2 μm, thermoelastic dissipation and anchor loss dominate and the *Q* factor increases with diameter, reaching ~3315 at a diameter of 2 μm. When the diameter further increases, the damping induced by surface nonidealities becomes more important and the *Q* factors dramatically decrease to ~10 at a diameter of 8 μm. Therefore, to achieve high-*Q* factors in 2D NEMS resonators a clean, smooth, and high-quality suspended membrane is the key for minimizing surface-induced energy loss.

We consistently aim for high *Q* factor in NEMS devices as it is critical for potential applications of MoS_2_ NEMS resonators in terms of sensitivity, frequency stability, and power efficiency. Specifically, a decreased *Q* factor can reduce the sensitivity of the resonator to external stimuli, affecting its performance in sensing applications. Lower *Q* factors may result in reduced frequency resolution, limiting the accuracy of RF signal processing and communication. Larger damping can also increase energy dissipation leading to higher power consumption and reduced energy efficiency. When the resonator continues to scale down, the surface nonidealities are less significant, and several techniques can further minimize energy dissipation and enhance the *Q* factor. For example, optimizing the environment by reducing the temperature and vacuum pressure^25,41,42^, applying external tensile strain to the suspended membrane^32^, performing vacuum thermal annealing after transfer^38^, and growing and transferring high-quality 2D materials with fewer material defects can significantly reduce damping and enhance the *Q* factor^49^. Furthermore, developing fabrication techniques that can reduce surface nonidealities is important for further optimizing the *Q* factor. For example, employing a stress-free transfer process with the assistance of poly(methyl methacrylate) (PMMA) and growing uniform high-quality 2D materials can facilitate the fabrication of a large-scale array of 2D NEMS resonators^29,50^. This approach could help reduce wrinkles and residues on the resonators to further enhance the *Q* factor, but could be more time-consuming. Polymers such as SU-8 can provide support when 2D materials are released, but wet chemical processes are used^16^. Therefore, there is a growing need to develop simple fabrication techniques for producing high-quality 2D NEMS resonators with clean and flat surfaces.

**Fig. 6:**
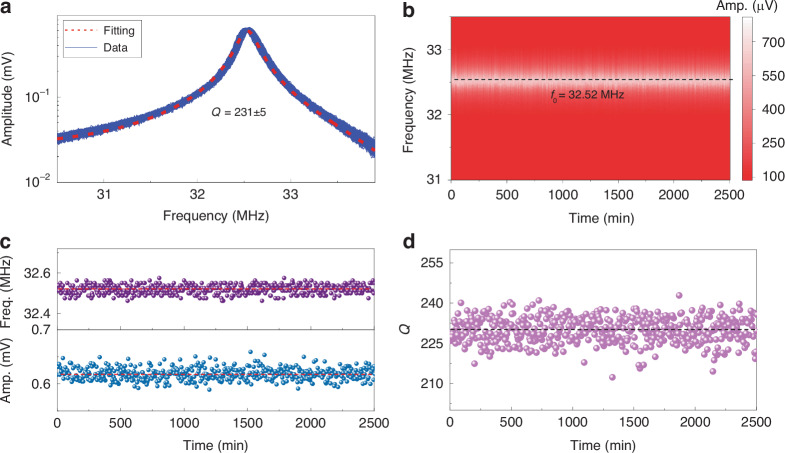
Long-term stability and reliability measurements of the MoS_2_ NEMS resonator Device 5-3 shown in Fig. 1a, with a diameter of 3 μm. **a** Summary of all the measured resonance curves for 2500 min at *V*_GS_ = 15 V and *v*_RF_ = 50 mV, showing high stability and repeatability over time. The red dashed line is the fitting to the average curve, with an extracted *Q* factor of 231 ± 5. **b** Color map of resonances in (**a**) varying with time, showing a very stable resonance frequency of ~32.52 MHz (black dashed line), with the amplitude shown in color scale. Extracted (**c**) resonance frequency and amplitude and (**d**) *Q* factor varying with time for resonances within (**a**)

Given that long-term stability and reliability are critical for commercial and industrial applications, we measure Device 5-3 in Fig. 1a over time and summarize how the frequency and *Q* factor change. Initially, the device is placed in a vacuum chamber and the system is continuously pumped to evacuate air within the cavity and maintain a stable vacuum pressure. Subsequently, we maintain a fixed electric driving force and record the resonances for 500 times with a 5-min time gap. The measured resonances exhibit robust and repeatable characteristics over time (Fig. 6a), with stable vibration amplitudes, resonance frequencies, and *Q* factors (Fig. 6b–d). This robust and reliable operation of 2D MoS_2_ NEMS resonators makes them highly promising for applications in sensing, RF signal processing, and computing units.

## Conclusions

In summary, we measure 52 2D MoS_2_ NEMS resonators with varying diameters from 1 μm to 8 μm, and achieve the highest *Q* factor of 3315 ± 115 for a resonator with a diameter of 2 μm. This is achieved through optimizing the device geometry by considering the effects of surface nonidealities on dissipation, the competition between different damping mechanisms, and optimizing the driving conditions by considering the strain-modulated dissipation model. We further demonstrate the long-term stability of the resonators. This study sheds light on the mechanisms behind the previously observed low *Q* factor in 2D NEMS resonators and provides clear guidelines for designing high-*Q* 2D NEMS resonators, which is important for their applications in sensing, RF signal processing, quantum engineering, memory, and computing.

## Supplementary information

SI_Markedup

## Data Availability

All the data needed to evaluate the conclusions in the paper are presented in the paper and/or the Supplementary Information. Other relevant data from this study are available from the corresponding author upon reasonable request.
