# Supplementary material for: Unveiling the tradeoff between device scale and surface nonidealities for an optimized quality factor at room temperature in 2D MoS2 nanomechanical resonators
Source: Microsyst Nanoeng. 2024 Sep 27;10:140. doi: 10.1038/s41378-024-00763-9 (PMC11427663; doi:10.1038/s41378-024-00763-9)
Supplement: Supplementary file 1 — SI_Markedup [file 41378_2024_763_MOESM1_ESM.pdf]

Supporting Information

Unveiling the Tradeoff between Device Scale and Surface Nonidealities for an Optimized Quality Factor at Room Temperature in 2D MoS_2 Nanomechanical Resonators

Pengcheng Zhang[1], Yueyang Jia[1], Shuai Yuan[1], Maosong Xie[1], Zuheng Liu[1], Hao Jia[2,*], Rui Yang[1,3,*]

[1]*University of Michigan–Shanghai Jiao Tong University Joint Institute, Shanghai Jiao Tong University, Shanghai 200240, China*

[2]*Shanghai Institute of Microsystem and Information Technology, Chinese Academy of Sciences, Shanghai 200050, China*

[3]*State Key Laboratory of Radio Frequency Heterogeneous Integration, Shanghai Jiao Tong University, Shanghai 200240, China*

[*]Corresponding author. Emails: hao.jia@mail.sim.ac.cn, rui.yang@sjtu.edu.cn

Table of Contents

S1. Fabrication and Measurement for 2D MoS_2 NEMS Resonators

S2. Scanning Electron Microscopy (SEM) and Atomic Force Microscopy (AFM) Measurements

S3. Raman Spectroscopy Measurements

S4. Models of Gate-Tunable Frequency and Quality (*Q*) Factor

S5. Summary of Measured Gate-Tunable Frequencies and *Q* Factors

S6. Finite Element Simulation of Surface Nonidealities on MoS_2 NEMS Resonators

S1. Fabrication and Measurement for 2D MoS_2 NEMS Resonators

The fabrication starts from lithographically defining the circular microtrenches and performing reactive ion etching (RIE) on an oxidized silicon wafer. The source/drain contact electrodes are then defined with photolithography and evaporation of chromium/gold, forming the substrate with microtrenches and electrodes. Finally, we exfoliate the 2D MoS_2 crystal onto polydimethylsiloxane (PDMS) stamps and transfer the 2D material onto the microtrenches and in contact with the electrodes using a dry transfer technique developed previously,[1] forming the suspended 2D MoS_2 NEMS resonator with contact electrodes for electrical driving.

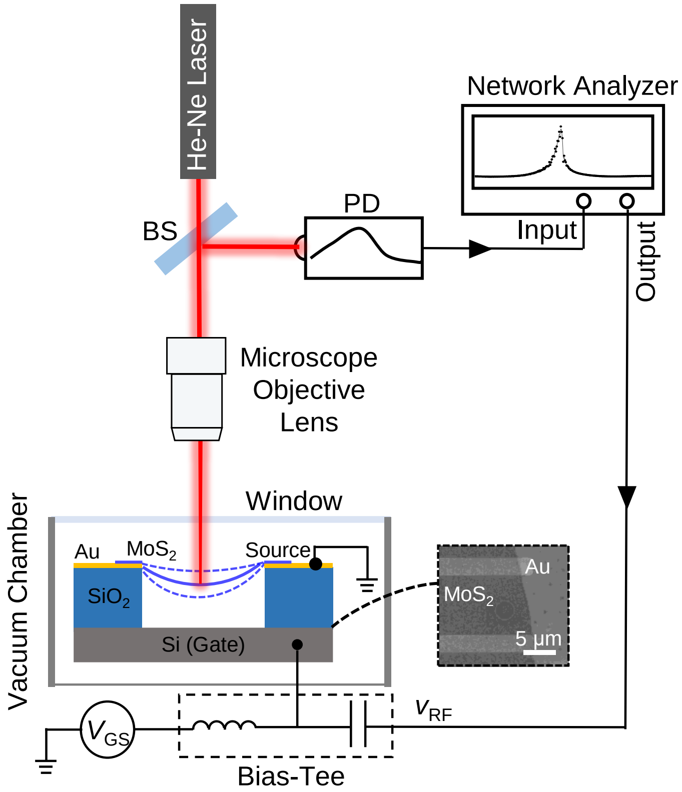

Figure S1. Schematic illustration of the optical interferometry measurement setup. The 633 nm He-Ne laser passes a beam splitter (BS), and is then focused onto the device through a 50× objective lens with window correction. The reflected beam, redirected by the BS, is focused onto a photodetector (PD) with its photoresponsivity over wavelength illustrated. The electronic signal is transmitted to a network analyzer. The device vibrates inside a vacuum chamber with a quartz

window at room temperature. *Inset*: the scanning electron microscopy (SEM) image of the same drumhead circular MoS_2 NEMS resonator "Device S1" as in Figure 4 in the Main Text.

The custom-built laser interferometry measurement system is illustrated in Figure S1. After wire bonding, the device is mounted in a vacuum chamber with optical window at room temperature. The v_{RF} is generated by a network analyzer and the V_{GS} is provided by a DC voltage source, which are combined through a bias tee and connected to the gate electrode to capacitively drive the resonator, with the contact electrode grounded. The mechanical oscillation is detected by a 633 nm laser. When the suspended membrane vibrates, the vacuum gap between the MoS_2 and the back gate will periodically change, leading to a change in the reflectance, which can be detected with the photodetector and then sent back to the network analyzer.[2] The thickness of the MoS_2 membrane is measured using a confocal microprobe Raman spectroscopy, and using AFM. The surface roughness of the MoS_2 is characterized by AFM and SEM. The material property is measured using PL and Raman spectroscopy.

S2. Scanning Electron Microscopy (SEM) and Atomic Force Microscopy (AFM) Measurements

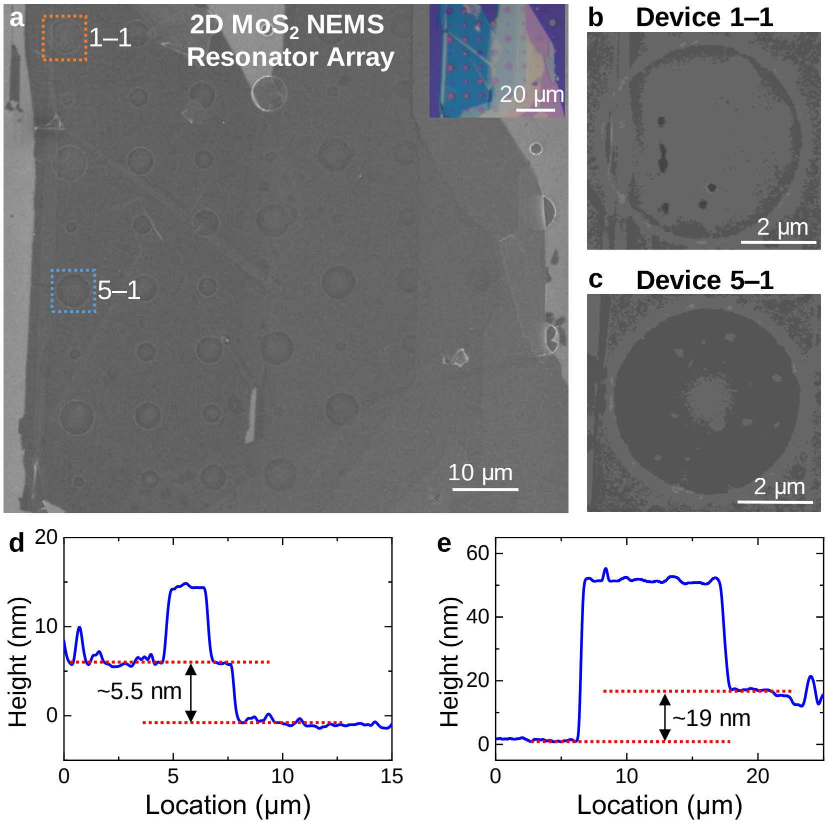

Figure S2. SEM and AFM Characterization of the array of 2D MoS_2 NEMS resonators shown in Figure 1 in the Main Text. (a) SEM image of the resonator array. *Inset*: optical image. (b) Zoom-in SEM image of Device 1–1 as shown by the orange dashed box in (a), showing many wrinkles and residues. (c) Zoom-in SEM image of Device 5–1 as shown by the blue dashed box in (a), showing less wrinkles and residues. (d–e) AFM line scans along the black dashed lines (d) "A" and (e) "B" as shown in Figure 1 in the Main Text.

We perform SEM and AFM characterization of the MoS_2 resonator array in Figure 1 in the Main Text (Figure S2). SEM images in Figure S2 a–c show surface nonidealities, and AFM line scans in Figure S2 d–e show the thickness (t) of the thin regions to be 5.5 nm, and the thick regions to be 19 nm.

S3. Raman Spectroscopy Measurements

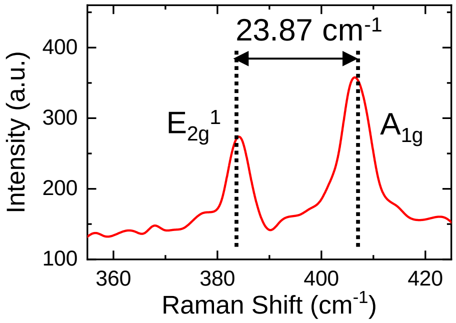

Figure S3. Raman spectrum of the same MoS_2 drumhead resonator "Device S5" as in Figure 5 in the Main Text. The difference between E_{2g}^1 and A_{1g} is 23.87 cm^{-1}, showing that it is few-layer MoS_2.

We can determine the thickness of monolayer, bilayer and trilayer MoS_2 using Raman spectroscopy, by analyzing the separation between the two peaks (E_{2g}^1 and A_{1g}).[3] However, for thicker material, the Raman peak separation is not deterministic. For example, the thickness of MoS_2 membrane for "Device S5" in Main Text is measured by Raman spectroscopy as shown in Figure S3, where the difference between E_{2g}^1 and A_{1g} peaks is 23.87 cm^{-1}, corresponding to few-layer MoS_2. For these thicker MoS_2 membrane, AFM measurements are then used, showing that the thickness of the MoS_2 membrane for "Device S5" is around 10 nm (Figure 5a–5c in the Main Text).

S4. Models of Gate-Tunable Frequency and Quality (Q) Factor

S4.1 Gate Tuning of Strain in Fully-Clamped 2D NEMS Resonators

When a 2D NEMS resonator is driven into motion, the mechanical oscillation of a linear oscillator can be modeled as a damped spring-mass system considering linear damping and a driving force $F(t)$, which follows the equation of motion:

$$\ddot{x}+\frac{\gamma}{m_{eff}}\dot{x}+\omega_0^2 x=\frac{F(t)}{m_{eff}}, \qquad \text{(S1)}$$

where x is the time-dependent displacement in the out-of-plane direction, ω_0 is the angular resonance frequency, $F(t) = F_{\text{drive}}\cdot\cos(\omega t)$ is the time-dependent external RF driving force, γ is the linear damping coefficient, and m_{eff} is the effective mass. We use a frequency tuning model for fully-clamped 2D circular drumhead NEMS resonators to extract the strain. When the DC gate voltage V_{GS} is applied, we can calculate the elastic energy stored in the stretched membrane E_{el} by:[4,5]

$$E_{el}=\int_0^{2\pi}d\theta\int_0^{R}rdr\left\{\underbrace{\frac{E_Y t}{2}\left[\varepsilon_{0,r}\left(\frac{\partial w}{\partial r}\right)^2+\frac{\varepsilon_{0,\theta}}{r^2}\left(\frac{\partial w}{\partial \theta}\right)^2\right]}_{potential}+\underbrace{\frac{t}{8}\frac{E_Y}{1-v^2}\left(\frac{\partial w}{\partial r}\right)^4}_{elongational}\right\}, \qquad \text{(S2)}$$

where R is the radius, t is the thickness, E_{Y} is the Young's modulus, v is the Poisson's ratio, $\varepsilon_{0,\text{r}}$ is the initial radial strain, $\varepsilon_{0,\theta}$ is the and initial tangential strain, and $w(r)$ is the deflection profile of the suspended material due to gate voltage. The first term of E_{el} corresponds to the effect of residual membrane stress and the potential energy, and the second term corresponds to the effect of elongation.

The electrostatic energy E_{es} stored in the fully-clamped resonator can be calculated by:

$$E_{es}=\frac{1}{2}C_g V_{GS}^2\,. \qquad \text{(S3)}$$

where C_{g} is the capacitance between the deflected membrane and the back gate.

The equilibrium position z_{e} when V_{GS} is applied can be solved by minimizing the total energy:

$$\frac{\partial\left(E_{es}-E_{el}\right)}{\partial z_e}=0\,. \tag{S4}$$

With z_e estimated, the total radial strain ε_r is obtained from the sum of the initial strain ε_0, and the strain generated by the deflection of the membrane:

$$\varepsilon_r=\frac{1+\varepsilon_{0,r}}{2R}\int_{-R}^{R}\sqrt{1+\left(\frac{\partial w}{\partial r}\right)^2}\,dr-1\,. \tag{S5}$$

S4.2 Gate Tuning of Resonance Frequency in Fully-Clamped 2D NEMS Resonators

The mode shape of the fundamental flexural mode resonance of a fully-clamped circular drumhead 2D NEMS resonator is given by $u_{\text{F-C}}(r)$ = $x_0J_0(2.405r/R)$, where x_0 is the maximum vibration amplitude, J_0 is the 0th-order Bessel function of the first kind: J_0 = 1-(2.405r/R)^2/4. The dynamic elastic energy and electrostatic energy at resonance can be estimated by substituting the mode shape into eq. S2:

$$\delta E_{el}=\frac{2.405^4\pi E_Y t\varepsilon_r}{16}x^2+\frac{2.405^8\pi E_Y t}{384\left(1-v^2\right)R^2}x^4\,. \tag{S6}$$

The resonance frequency in the linear resonance regime can be obtained by $f_0=\frac{1}{2\pi}\sqrt{\frac{k_{eff}}{m_{eff}}}$, where k_{eff} is the effective dynamic spring constant, and m_{eff} is the effective mass. The k_{eff} is given by:

$$k_{eff}=\frac{\partial^2\delta E_{el}}{\partial x_0^2}+\frac{\partial^2 E_{es}}{\partial z_e^2}\approx\frac{2.405^4E_Y t\varepsilon_r}{8}-\frac{\epsilon_0\pi R^2}{3g^3}V_{GS}{}^2\,. \tag{S7}$$

The m_{eff} of fundamental-mode resonance can be derived from the mode shape:

$$m_{eff}=\rho t\int_0^{2\pi}\int_0^{R}\left(J_0\left(\frac{2.4}{R}r\right)\right)^2 r\,dr\,d\theta=0.25\rho t\pi R^2\,. \tag{S8}$$

Thus, the resonance frequency f_0 tuning by V_{GS} for circular membrane NEMS resonators can be given by:

$$f_0=\frac{1}{2\pi}\sqrt{\frac{2.405^4E_Y\varepsilon_r}{2\rho R^2}-\frac{\epsilon_0}{0.75\rho t g^3}V_{GS}{}^2}\,. \tag{S9}$$

S4.3 Strain-Modulated Linear Damping Mechanism in 2D NEMS Resonators

Considering different dissipation mechanisms, the total quality factor Q can be expressed as a combination of intrinsic damping factors and extrinsic damping factors:[5]

$$Q^{-1} = Q_{intrinsic}^{-1} + Q_{extrinsic}^{-1} . \tag{S10}$$

For the part of intrinsic damping that is due to the flexure and elongation of a stretched 2D membrane, it has been previously studied as thermoelastic damping for fully-clamped circular resonators, with the elongational damping taking the dominant role:

$$Q_{TED}^{-1} = \frac{E_{elong}}{E_{pot}} \delta_{elong} , \tag{S11}$$

where δ is the loss angle, E_{pot} is the potential energy, and E_{elong} is the elongational energy of the oscillating 2D membrane. Dissipation mechanisms in devices depend on their own mismatch in the phase space. The Young's modulus of the material has a complex form: $E_Y^* = E_Y(1+i\delta)$, where δ is the loss angle. In 2D NEMS, the loss angle is δ:

$$\delta = \frac{\Delta E}{2\pi E} . \tag{S12}$$

The energy in a fully-clamped circular drumhead 2D MoS_2 resonator under strain include the energy from the effect of elongation and residual membrane stress (potential energy), as shown by the different terms in eq. S2:

$$\begin{cases} E_{elong} = \dfrac{2.405^8 \pi E_Y t}{384\left(1-v^2\right)R^2} {x_0}^4 \\ E_{pot} = \dfrac{2.405^4 \pi E_Y t \varepsilon_r}{16} {x_0}^2 \end{cases} , \tag{S13}$$

Then we plug them into eq. S12, and get the thermoelastic dissipation for fully-clamped devices:

$$Q_{lin,TED}^{-1} = \frac{5.576 {x_0}^2}{\varepsilon_r\left(V_{GS}\right)R^2\left(1-v^2\right)} \delta , \tag{S14}$$

where δ is also a fitting parameter, $\varepsilon_r\left(V_{GS}\right)$ is the strain that can be tuned by V_{GS}, and x_0 is proportional to $|V_{GS} \cdot v_{RF}|$ when the dissipation mechanism is dominated by linear damping.

S5. Summary of Measured Gate-Tunable Frequencies and *Q* Factors

When a 2D NEMS resonator is driven into motion, the mechanical oscillation of a linear oscillator can be modeled as a damped spring-mass system considering linear damping and a driving force $F(t)$, which follows the equation of motion:

$$\ddot{x} + \frac{\gamma}{m_{eff}}\dot{x} + \omega_0^2 x = \frac{F(t)}{m_{eff}}, \tag{S15}$$

where x is the time-dependent displacement in the out-of-plane direction, ω_0 is the angular resonance frequency, $F(t) = F_{\text{drive}}\cdot\cos(\omega t)$ is the time-dependent external RF driving force, γ is the linear damping coefficient, and m_{eff} is the effective mass. By using the method of harmonic balance, we obtain the solution:

$$\left|x_0(\omega)\right|^2 = \frac{\left(\frac{F_{drive}}{m_{eff}}\right)^2}{\left(\omega^2-\omega_0^2\right)^2+\left(\frac{\gamma}{m_{eff}}\omega\right)^2}. \tag{S16}$$

The *Q* factor for each resonance is extracted by fitting to the solution for equation, with Figure S4 showing the examples of resonance fitting for the array of NEMS resonators in Figure 1 in the Main Text.

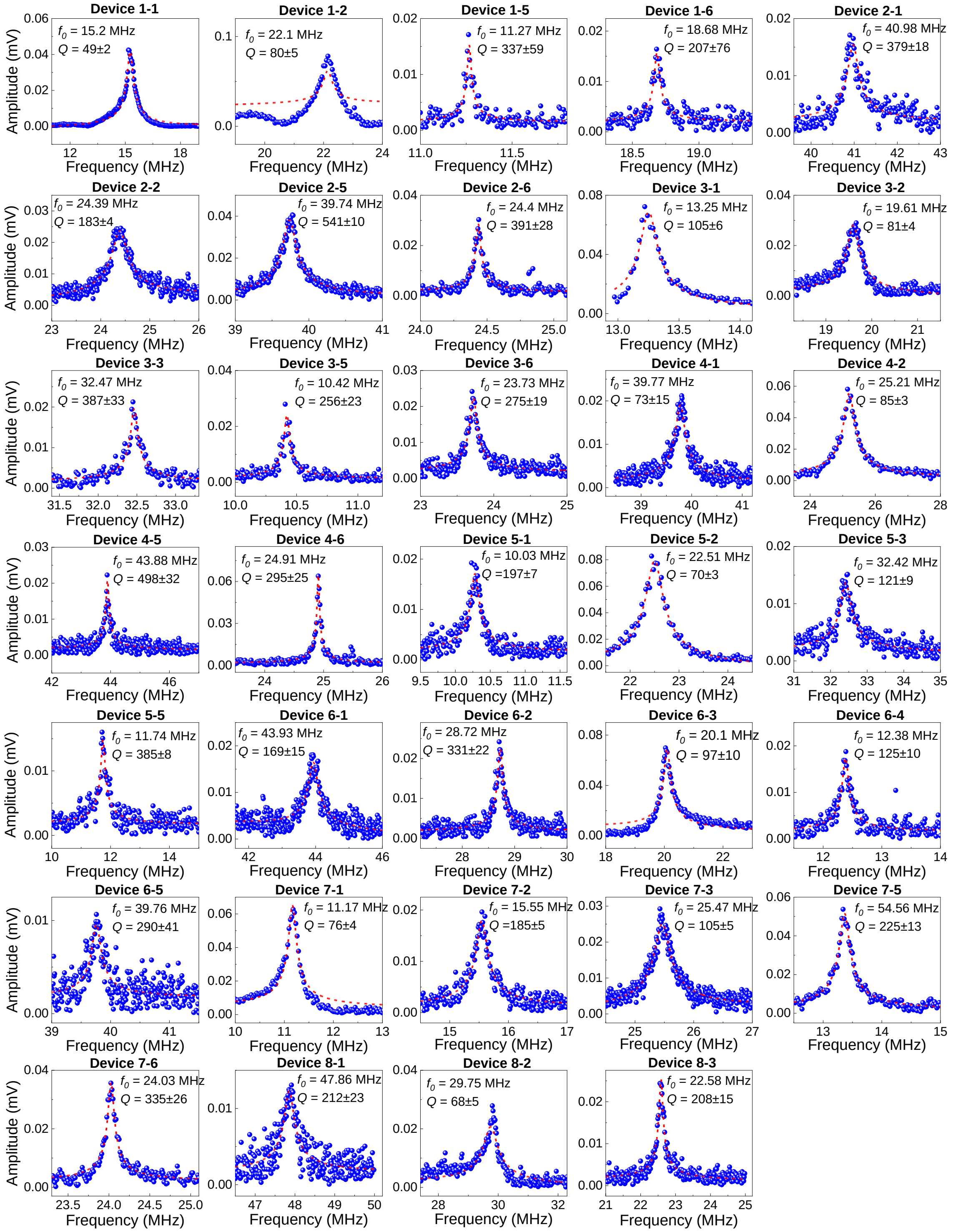

Figure S4. Measured resonances and extracted Q factors for MoS_2 drumhead NEMS resonators for the resonator array in Figure 1 in the Main Text, at V_{GS}=1 V and v_{RF}= 20 mV.

Table S1. List of the single isolated MoS_2 drumhead NEMS resonators and their parameters.

Device Number	D (µm)	t	$\varepsilon_{0,r}$ (%)	δ	Q_{max}	Wrinkle/ Bubble	Residue
**S1** (Figure 2a)	2	2 layer	0.1	0.007	3105	×	×
**S2** (Figure 3a)	1	1 layer	0.05	0.008	1051	×	×
**S3** (Figure 4a)	6	2 layers	×	0.09	60	✓	✓
**S4** (Figure 4l)	4	1 layer	×	0.1	81	✓	×
**S5-"a"** (Figure 5c)	3	10 nm	×	0.05	129	✓	✓
**S5-"b"** (Figure 5b)	3	10 nm	×	×	×	✓	×
**S5-"c"** (Figure 5b)	3	10 nm	×	0.06	188	✓	×
**S5-"d"** (Figure 5d)	3	10 nm	0.02	0.02	572	×	×
**S5-"e"** (Figure 5d)	3	10 nm	0.02	0.03	351	✓	×
**S6** (Figure S5)	8	3 nm	×	0.11	10	✓	×
**S7** (Figure S6)	8	3.5 nm	×	0.15	15	✓	✓
**S8** (Figure S7)	6	12.5 nm	0.07	0.08	60	✓	×
**S9** (Figure S8)	4	7.5 nm	0.15	0.07	99	✓	×
**S10** (Figure S9)	4	4.5 nm	0.13	0.06	100	✓	×
**S11** (Figure S10)	4	5 nm	0.009	0.06	100	✓	×
**S12** (Figure S11)	3	4.5 nm	0.007	0.02	696	✓	✓
**S13** (Figure S12)	3	10 nm	0.005	0.03	601	×	✓
**S14** (Figure S13)	2	5.5 nm	0.08	0.01	1211	×	✓
**S15** (Figure S14)	2	7 nm	0.003	0.008	1128	✓	×
**S16** (Figure S15)	2	7 nm	0.005	0.018	1779	×	×
**S17** (Figure S16)	1	10.5 nm	0.015	0.01	661	×	✓
**S18** (Figure S17)	1	11 nm	0.019	0.008	980	×	✓

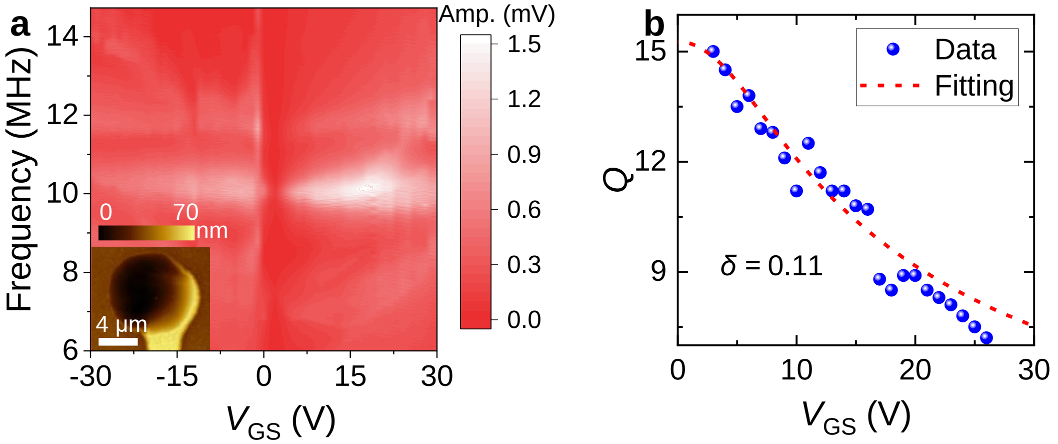

Figure S5. Measured resonance frequency and Q tuned by gate voltage for a MoS_2 drumhead NEMS resonator with diameter (D) of 8 μm named "Device S6". (a) Measured frequency tuning characteristics with the resonance amplitude shown in color scale, by varying V_{GS} and with v_{RF} fixed at 20 mV. *Inset*: AFM image with the height in color scale showing the roughness of surface for the suspended MoS_2 membrane. (b) Measured gate tuning of Q, where the Q at each V_{GS} is extracted from fitting the resonance in (a). The blue dots are the experimental data, and the red dashed line is the model fitting, with δ of 0.11 extracted.

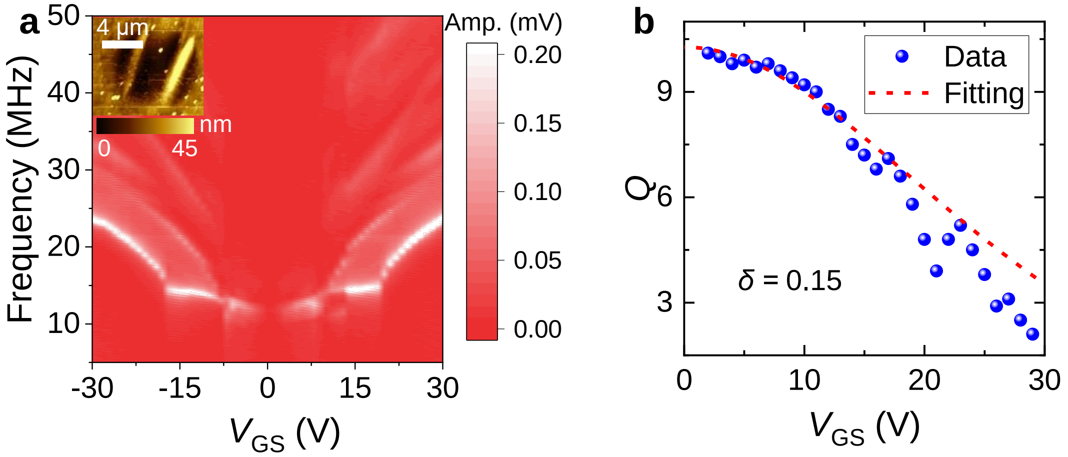

Figure S6. Measured resonance frequency and Q tuned by gate voltage for a MoS_2 drumhead NEMS resonator with diameter of 8 μm named "Device S7". (a) Measured frequency tuning characteristics with the resonance amplitude shown in color scale, by varying V_{GS} and with v_{RF} fixed at 20 mV. *Inset*: AFM image with the height in color scale showing the roughness of surface for the suspended MoS_2 membrane. (b) Measured gate tuning of Q, where the Q at each V_{GS} is extracted from fitting the resonance in (a). The blue dots are the experimental data, and the red dashed line is the model fitting, with δ of 0.15 extracted.

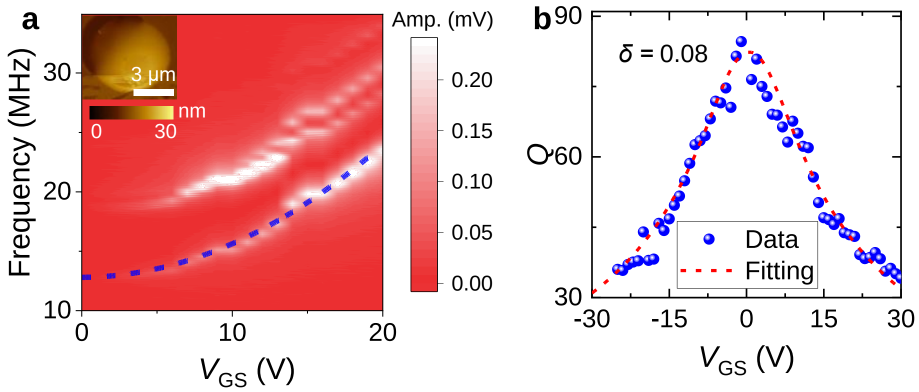

Figure S7. Measured resonance frequency and Q factor tuned by gate voltage for a MoS_2 drumhead NEMS resonator with diameter of 6 µm named "Device S8". (a) Measured frequency tuning characteristics with the resonance amplitude shown in color scale, by varying V_{GS} and with v_{RF} fixed at 20 mV. *Inset*: AFM image with the height in color scale showing the roughness of surface for the suspended MoS_2 membrane. (b) Measured gate tuning of Q factor, where the Q factor at each V_{GS} is extracted from fitting the resonance in (a). The blue dots are the experimental data, and the red dashed line is the model fitting, with δ of 0.08 extracted.

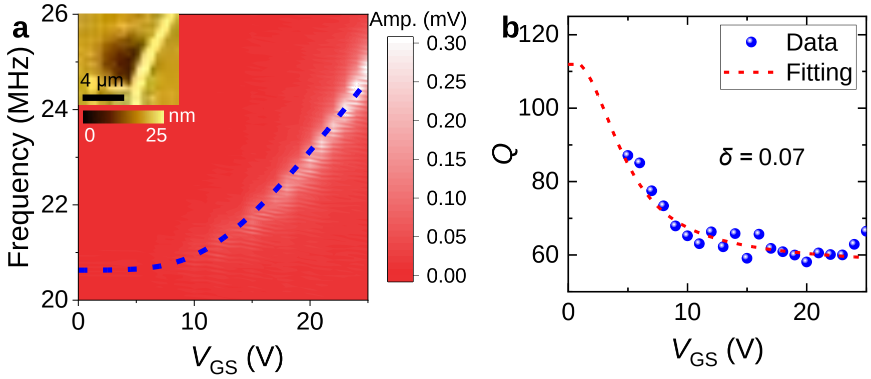

Figure S8. Measured resonance frequency and Q factor tuned by gate voltage for a MoS_2 drumhead NEMS resonator with diameter of 4 µm named "Device S9". (a) Measured frequency tuning characteristics with the resonance amplitude shown in color scale, by varying V_{GS} and with v_{RF} fixed at 20 mV. *Inset*: AFM image with the height in color scale showing the roughness of surface for the suspended MoS_2 membrane. (b) Measured gate tuning of Q factor, where the Q factor at each V_{GS} is extracted from fitting the resonance in (a). The blue dots are the experimental data, and the red dashed line is the model fitting, with δ of 0.07 extracted.

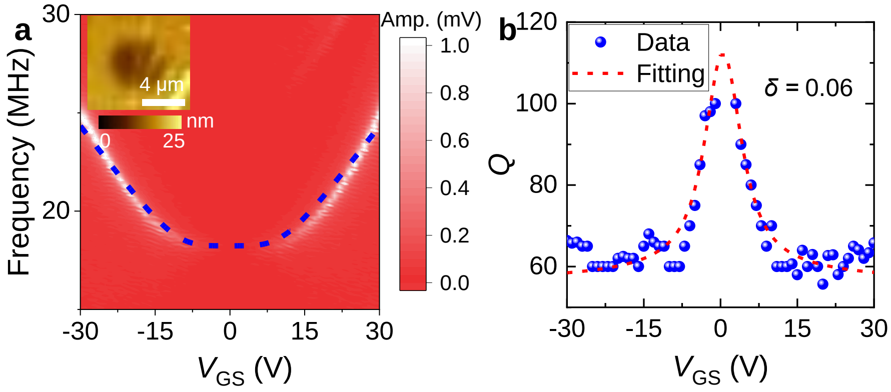

Figure S9. Measured resonance frequency and Q factor tuned by gate voltage for a MoS_2 drumhead NEMS resonator with diameter of 4 μm named "Device S10". (a) Measured frequency tuning characteristics with the resonance amplitude shown in color scale, by varying V_{GS} and with v_{RF} fixed at 20 mV. *Inset*: AFM image with the height in color scale showing the roughness of surface for the suspended MoS_2 membrane. (b) Measured gate tuning of Q factor, where the Q factor at each V_{GS} is extracted from fitting the resonance in (a). The blue dots are the experimental data, and the red dashed line is the model fitting, with δ of 0.06 extracted.

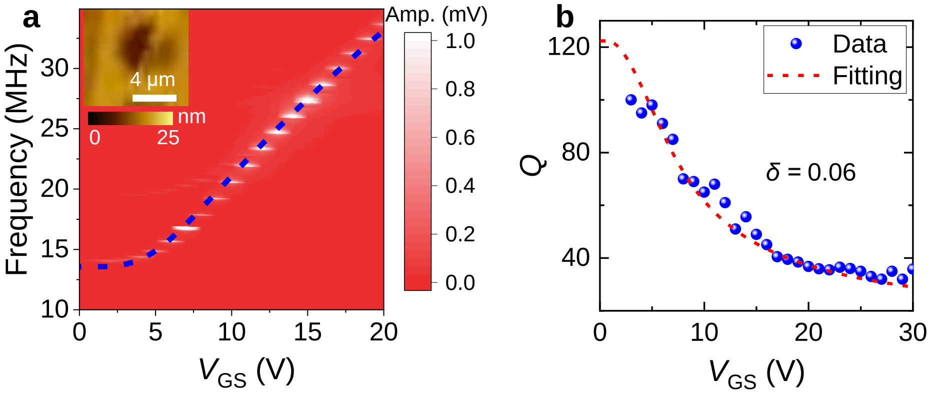

Figure S10. Measured resonance frequency and Q factor tuned by gate voltage for a MoS_2 drumhead NEMS resonator with diameter of 4 μm named "Device S11". (a) Measured frequency tuning characteristics with the resonance amplitude shown in color scale, by varying V_{GS} and with v_{RF} fixed at 20 mV. *Inset*: AFM image with the height in color scale showing the roughness of surface for the suspended MoS_2 membrane. (b) Measured gate tuning of Q factor, where the Q factor at each V_{GS} is extracted from fitting the resonance in (a). The blue dots are the experimental data, and the red dashed line is the model fitting, with δ of 0.06 extracted.

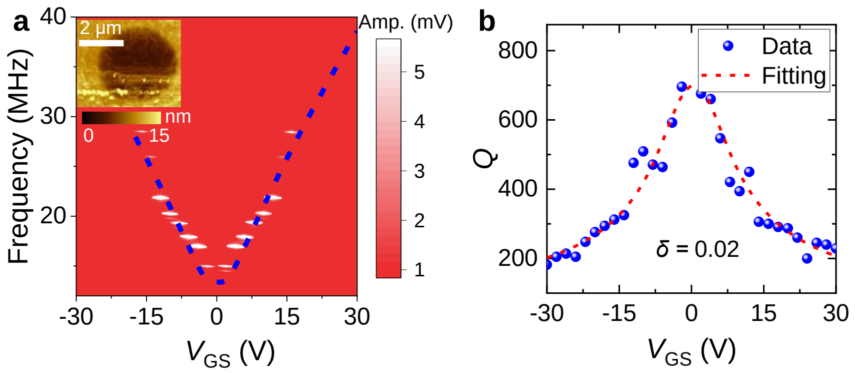

Figure S11. Measured resonance frequency and Q factor tuned by gate voltage for a MoS_2 drumhead NEMS resonator with diameter of 3 μm named "Device S12". (a) Measured frequency tuning characteristics with the resonance amplitude shown in color scale, by varying V_{GS} and with v_{RF} fixed at 20 mV. *Inset*: AFM image with the height in color scale showing the roughness of surface for the suspended MoS_2 membrane. (b) Measured gate tuning of Q factor, where the Q factor at each V_{GS} is extracted from fitting the resonance in (a). The blue dots are the experimental data, and the red dashed line is the model fitting, with δ of 0.02 extracted.

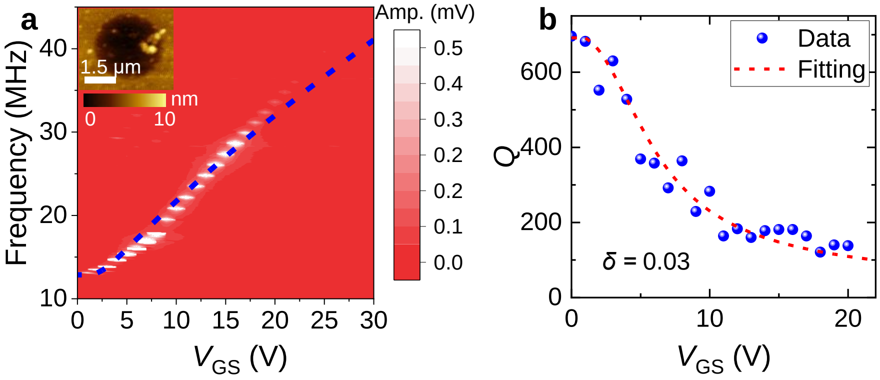

Figure S12. Measured resonance frequency and Q factor tuned by gate voltage for a MoS_2 drumhead NEMS resonator with diameter of 3 μm named "Device S13". (a) Measured frequency tuning characteristics with the resonance amplitude shown in color scale, by varying V_{GS} and with v_{RF} fixed at 20 mV. *Inset*: AFM image with the height in color scale showing the roughness of surface for the suspended MoS_2 membrane. (b) Measured gate tuning of Q factor, where the Q factor at each V_{GS} is extracted from fitting the resonance in (a). The blue dots are the experimental data, and the red dashed line is the model fitting, with δ of 0.03 extracted.

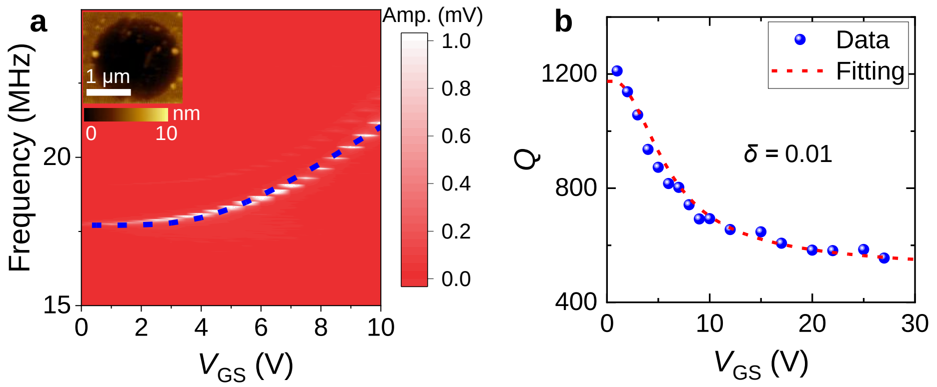

Figure S13. Measured resonance frequency and Q factor tuned by gate voltage for a MoS_2 drumhead NEMS resonator with diameter of 2 μm named "Device S14". (a) Measured frequency tuning characteristics with the resonance amplitude shown in color scale, by varying V_{GS} and with v_{RF} fixed at 20 mV. *Inset*: AFM image with the height in color scale showing the roughness of surface for the suspended MoS_2 membrane. (b) Measured gate tuning of Q factor, where the Q factor at each V_{GS} is extracted from fitting the resonance in (a). The blue dots are the experimental data, and the red dashed line is the model fitting, with δ of 0.01 extracted.

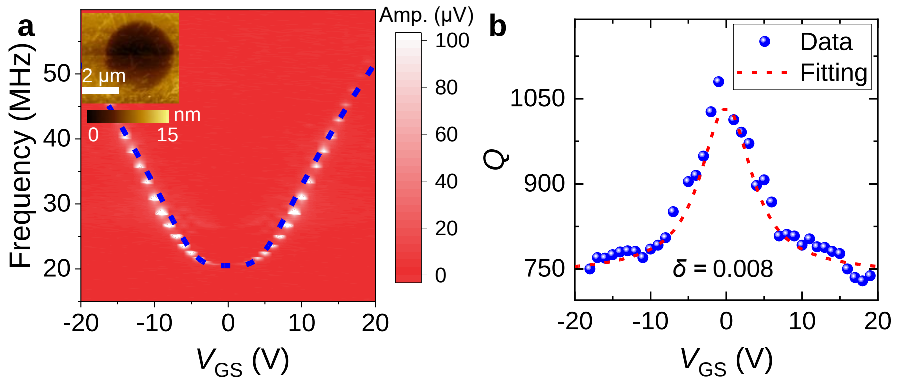

Figure S14. Measured resonance frequency and Q factor tuned by gate voltage for a MoS_2 drumhead NEMS resonator with diameter of 2 μm named "Device S15". (a) Measured frequency tuning characteristics with the resonance amplitude shown in color scale, by varying V_{GS} and with v_{RF} fixed at 20 mV. *Inset*: AFM image with the height in color scale showing the roughness of surface for the suspended MoS_2 membrane. (b) Measured gate tuning of Q factor, where the Q factor at each V_{GS} is extracted from fitting the resonance in (a). The blue dots are the experimental data, and the red dashed line is the model fitting, with δ of 0.008 extracted.

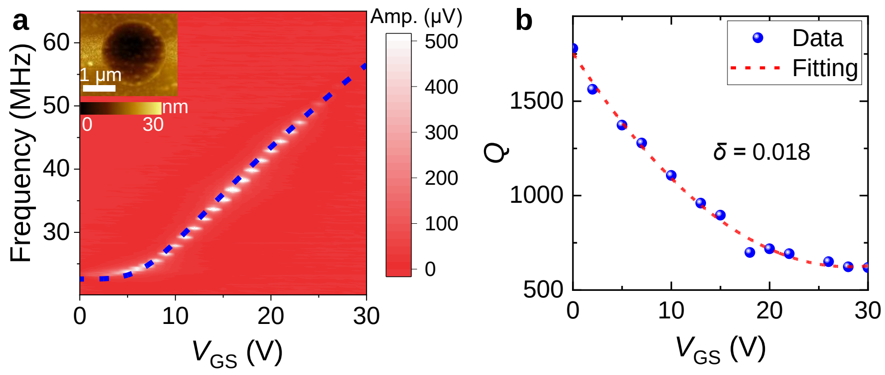

Figure S15. Measured resonance frequency and Q factor tuned by gate voltage for a MoS_2 drumhead NEMS resonator with diameter of 2 μm named "Device S16". (a) Measured frequency tuning characteristics with the resonance amplitude shown in color scale, by varying V_{GS} and with v_{RF} fixed at 20 mV. *Inset*: AFM image with the height in color scale showing the roughness of surface for the suspended MoS_2 membrane. (b) Measured gate tuning of Q factor, where the Q factor at each V_{GS} is extracted from fitting the resonance in (a). The blue dots are the experimental data, and the red dashed line is the model fitting, with δ of 0.018 extracted.

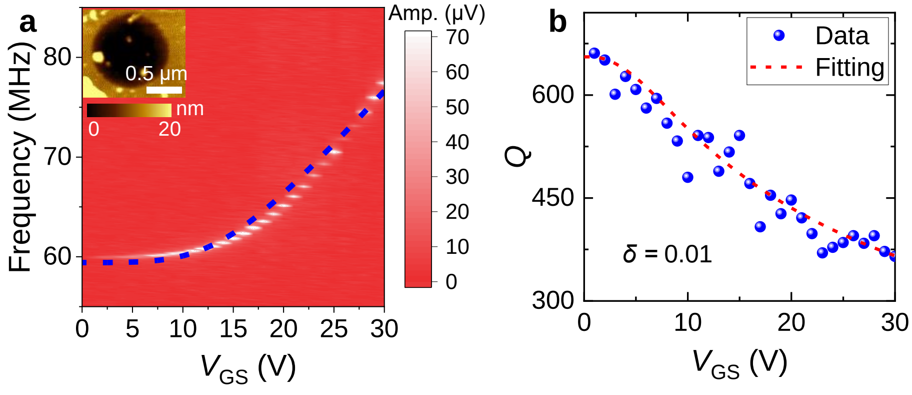

Figure S16. Measured resonance frequency and Q factor tuned by gate voltage for a MoS_2 drumhead NEMS resonator with diameter of 1 μm named "Device S17". (a) Measured frequency tuning characteristics with the resonance amplitude shown in color scale, by varying V_{GS} and with v_{RF} fixed at 20 mV. *Inset*: AFM image with the height in color scale showing the roughness of surface for the suspended MoS_2 membrane. (b) Measured gate tuning of Q factor, where the Q factor at each V_{GS} is extracted from fitting the resonance in (a). The blue dots are the experimental data, and the red dashed line is the model fitting, with δ of 0.01 extracted.

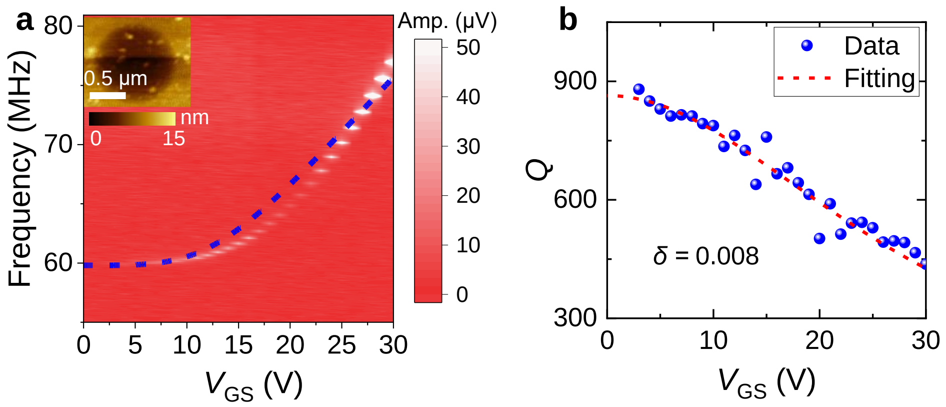

Figure S17. Measured resonance frequency and Q factor tuned by gate voltage for a MoS_2 drumhead NEMS resonator with diameter of 1 μm named "Device S18". (a) Measured frequency tuning characteristics with the resonance amplitude shown in color scale, by varying V_{GS} and with v_{RF} fixed at 20 mV. *Inset*: AFM image with the height in color scale showing the roughness of surface for the suspended MoS_2 membrane. (b) Measured gate tuning of Q factor, where the Q factor at each V_{GS} is extracted from fitting the resonance in (a). The blue dots are the experimental data, and the red dashed line is the model fitting, with δ of 0.008 extracted.

S6. Finite Element Simulation of Surface Nonidealities on MoS_2 NEMS Resonators

To investigate the effects of surface nonidealities on MoS_2 NEMS resonators, we conduct simulations using finite element method (FEM), for the resonators with residue (Figure S18), wrinkles at the center of the suspended membrane (Figure S19), and wrinkles at the edge of the suspended membrane (Figure S20). These surface nonidealities introduce nonuniform resonance mode shapes, particularly at higher applied V_{GS} values (Figures S18a, S19a, and S20a). For devices with residue, we observe peculiar frequency tuning tendencies, especially at large V_{GS}, which could not be well fitted by the tuning model with a consistent trend (Figure S18b). In addition, the second mode show very similar frequencies as the first mode. In contrast, the normal resonator without the residue can have regular frequency tuning characteristics that is well fitted by the model. Furthermore, for resonators with wrinkles, whether the wrinkles are at the center or the edge of the suspended membrane, the resonators exhibit much lower tuning ranges and unconventional frequency tuning characteristics (Figures S19b and S20b). We also simulate the effects of driving force and Q factor on resonance characteristics for a device with wrinkle (Figure S20c): at a same Q factor, the device with a larger driving force shows a much more significant 2nd mode; while at the same driving force, the device with a smaller Q factor shows a smaller selectivity for the 1st and 2nd modes.

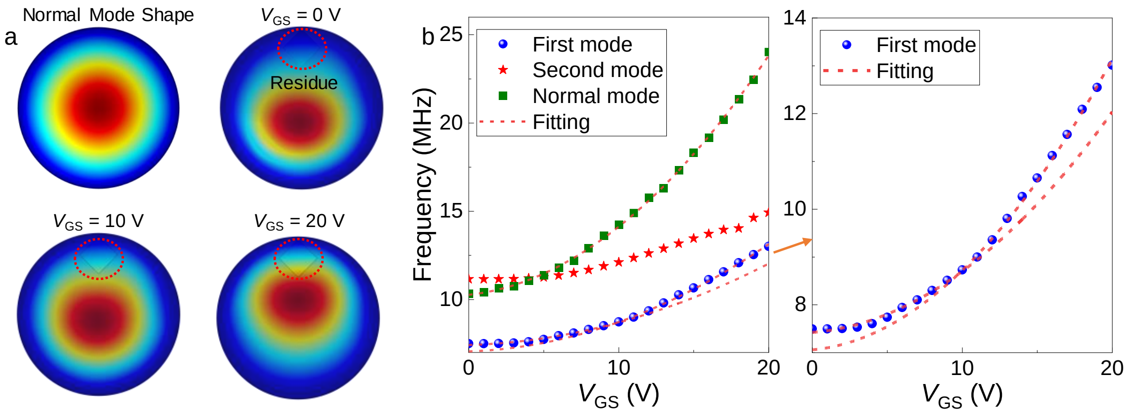

Figure S18. Finite element simulation of a 2D MoS_2 NEMS resonator with residue. (a) Simulated first flexural mode shape at different V_{GS} of 0 V, 10 V and 20 V. The red dashed lines illustrate the attached residue on the suspended membrane. The thickness, diameter, and initial vacuum gap of 2D MoS_2 resonator are 2 nm, 6 μm, and 600 nm, respectively. (b) Simulated frequency tuning

characteristics for the first and second modes, and comparison with the normal resonator without residue on the surface. The zoom-in view of the resonance frequencies and the fitting is also shown.

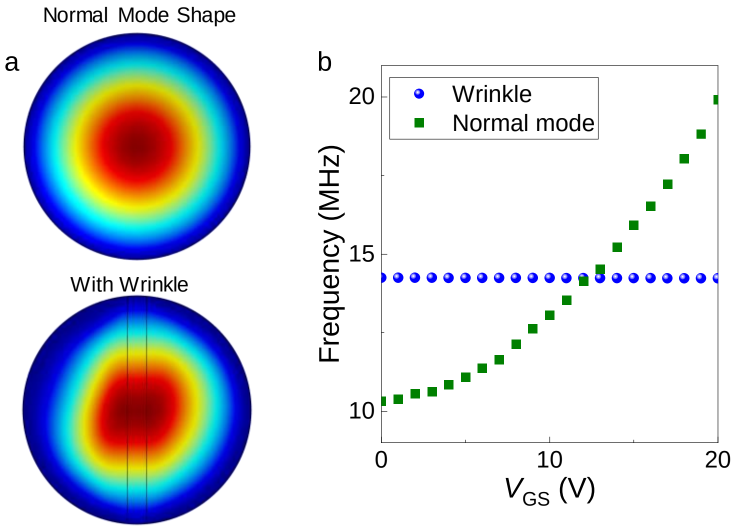

Figure S19. Finite element simulation of a 2D MoS_2 NEMS resonator with a wrinkle at the center of the suspended membrane. (a) Simulated first flexural mode shape at V_{GS} of 10 V. The thickness, diameter, and initial vacuum gap of the 2D MoS_2 resonator are 2 nm, 8 μm, and 600 nm, respectively. (b) Simulated frequency tuning characteristics for the first mode, in comparison with that for a normal resonator without the wrinkle.

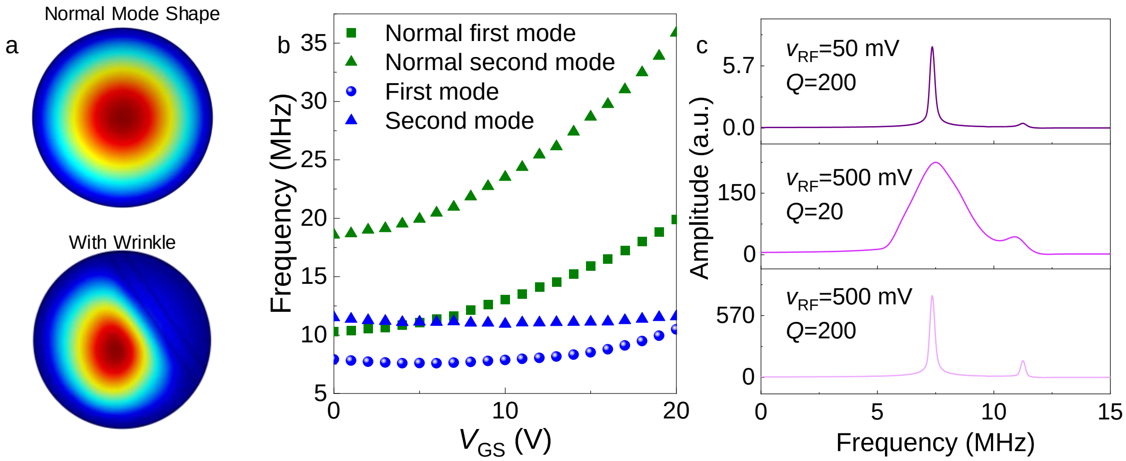

Figure S20. Finite element simulation of a 2D MoS_2 NEMS resonator with a wrinkle at the edge of the suspended membrane. (a) Simulated first flexural mode shape at V_{GS} of 10 V. The thickness, diameter, and initial vacuum gap of the 2D MoS_2 resonator are 2 nm, 8 μm, and 600 nm, respectively. (b) Simulated frequency tuning characteristics for the first mode, in comparison with that for a normal resonator without the wrinkle. (c) Simulated resonance responses for both 1st and 2nd modes for the wrinkled device in (a), by varying v_{RF} and Q factor.

References

1. Yang, R.; Zheng, X.; Wang, Z.; Miller, C. J.; Feng, P. X.-L. Multilayer MoS_2 Transistors Enabled by a Facile Dry-Transfer Technique and Thermal Annealing. *J. Vac. Sci. Technol. B* **2014**, *32*, 061203.

2 Lee, J.; Wang, Z.; He, K.; Yang, R.; Shan, J.; Feng, P. X. L. Electrically Tunable Single- and Few-Layer MoS_2 Nanoelectromechanical Systems with Broad Dynamic Range. *Sci. Adv*. **2018**, *4* (3), eaao6653.

3 Li, H; Zhang, Q.; Yap, C. C. R., Tay, B. K.; Edwin, T. H. T.; Olivier, A.; Baillargeat, D. From Bulk to Monolayer MoS_2: Evolution of Raman Scattering. *Adv. Funct. Mater.* **2012**, *22* (7), 1385-1390.

4 Schomburg, W. K. Membranes. In *Introduction to Microsystem Design*; Springer-Verlag, 2011.

5 Zhang, P.; Jia, Y.; Xie, M.; Liu, Z.; Sheng, S.; Wei, J.; Yang, R. Strain-Modulated Dissipation in Two-Dimensional Molybdenum Disulfide Nanoelectromechanical Resonators. *ACS Nano* **2022**, *16*, 2261–2270.
